# Endoscopic ultrasound-guided hepaticoduodenostomy for the management of iatrogenic bile duct injury related to cholecystectomy: a report of two cases

**DOI:** 10.1055/a-2690-1827

**Published:** 2025-09-09

**Authors:** Antoine Assaf, Jean-Philippe Ratone, Solene Hoibian, Yanis Dahel, Victor Garbay, Marc Giovannini, Fabrice Caillol

**Affiliations:** 1 56181 Department of Hepatogastroenterology, Paoli-Calmettes Institute, Marseille, France

The management of bile duct injuries (BDIs) involves endoscopic and radiologic interventions, with surgery reserved for complex cases 1 . We present two cases of BDI managed with endoscopic ultrasound-guided hepaticoduodenostomy (EUS-HDS).

**Case 1:** A 77-year-old patient underwent a cholecystectomy complicated by an injury to the common bile duct. A surgical choledochoduodenostomy was performed. One year later, the patient developed cholestasis due to a strictured anastomosis. Endoscopic retrograde cholangiopancreatography (ERCP) and percutaneous biliary drainage failed to traverse the anastomosis. EUS-hepaticogastrostomy was performed. Two weeks later, a nasobiliary catheter through the hepaticogastrostomy was used to distend the common hepatic duct (CHD) with normal saline. Linear EUS, from the duodenal bulb, enabled CHD puncture. A guidewire was advanced into the hepatic duct, followed by insertion of a 6-Fr cystotome and a 4-cm covered biliary stent ( Video 1 ). No complications occurred. The stent was later replaced with double-pigtail stents, exchanged every three months, over one year.

Endoscopic ultrasound-guided choledochoduodenostomy procedure for the management of a post-cholecystectomy bile duct injury after failed endoscopic retrograde cholangiopancreatography and percutaneous biliary drainage.Video 1

**Case 2:** A 75-year-old patient had a BDI (Strasberg D) during cholecystectomy, managed with surgical hepaticoduodenal anastomosis. Six months later, cholestasis and dilation of right hepatic ducts (RHDs) were noted ( Fig. 1 ). During endoscopic retrograde cholangiopancreatography, cannulation of the surgical anastomosis revealed exclusive drainage of the left hepatic ducts. A percutaneous drain with cholangioscopic assistance also failed to traverse the anastomosis. The RHDs were likely excluded. A percutaneous drain was placed for RHD distension. Under EUS guidance, the RHDs were punctured with a 19G needle. The hepaticoduodenal tract was dilated with a cystotome and a 6-mm balloon. Two double-pigtail stents were subsequently placed through the hepatobiliary tract orifice and exchanged every three months for one year ( Fig. 2 ).

**Fig. 1:**
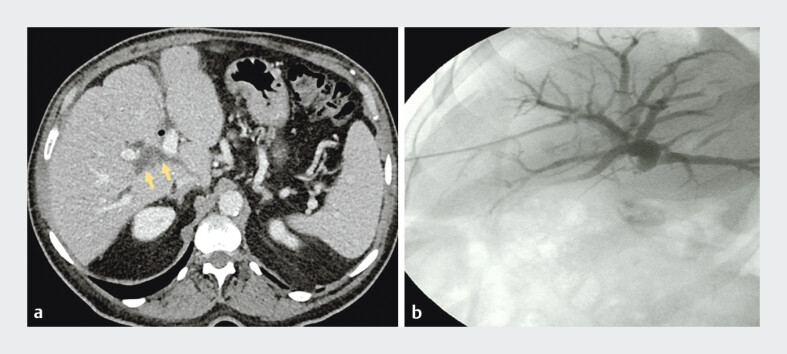
**a** Abdominal computed tomography image showing isolated right hepatic duct dilation. **b** Cholangiography during percutaneous biliary drainage showing dilation of the right hepatic ducts, which are excluded from the remaining biliary tree.

**Fig. 2:**
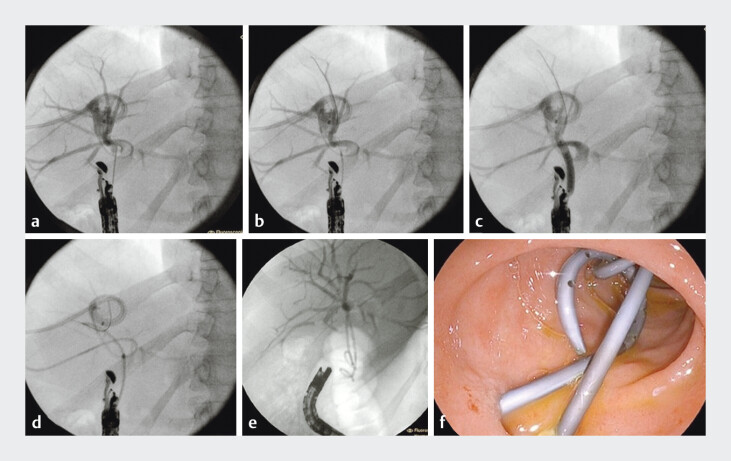
**a** Right bile duct puncture via a 19G fine-needle aspiration needle. **b** Fistula dilation using a 6-Fr cystotome. **c** Fistula dilation with a 6-mm biliary dilation balloon. **d** Insertion of the first double-pigtail plastic stent. **e** Fluoroscopic view of two double plastic stents through the hepaticoduodenal anastomosis. **f** Endoscopic view of the plastic stents in place.

We presented two cases of successful bile duct reconstruction using EUS-HDS after failed endoscopic and percutaneous interventions. This technique offers a minimally invasive alternative to surgical reconstruction in complex BDI. Larger series are still needed to assess the role of EUS-HDS in this indication.

Endoscopy_UCTN_Code_TTT_1AQ_2AZ

## Notes

Endoscopy E-Videos https://eref.thieme.de/e-videos
*E-Videos* is an open access online section of the journal *Endoscopy* , reporting on interesting cases and new techniques in gastroenterological endoscopy. All papers include a high-quality video and are published with a Creative Commons CC-BY license. Endoscopy E-Videos qualify for HINARI discounts and waivers and eligibility is automatically checked during the submission process. We grant 100% waivers to articles whose corresponding authors are based in Group A countries and 50% waivers to those who are based in Group B countries as classified by Research4Life (see: https://www.research4life.org/access/eligibility/ ). This section has its own submission website at https://mc.manuscriptcentral.com/e-videos .
